# Iron Impregnated Activated Carbon as an Efficient Adsorbent for the Removal of Methylene Blue: Regeneration and Kinetics Studies

**DOI:** 10.1371/journal.pone.0122603

**Published:** 2015-04-07

**Authors:** Irfan Shah, Rohana Adnan, Wan Saime Wan Ngah, Norita Mohamed

**Affiliations:** School of Chemical Sciences, Universiti Sains Malaysia, 11800 Penang, Malaysia; Massachusetts Institute of Technology, UNITED STATES

## Abstract

In this study, iron impregnated activated carbon (FeAC) was synthesized following an oxidation and iron impregnation of activated carbon (AC). Both the AC and FeAC were characterized by pH_*ZPC*_ and FTIR spectroscopy. The removal of Methylene Blue (MB) by AC and FeAC was examined under various experimental conditions. The FeAC showed up to 95% (higher than AC) MB removal in the pH range of 7–10. Although the reaction kinetics was pseudo–second order, the overall rate was controlled by a number of processes such as film diffusion, pore diffusion and intraparticle diffusion. The activation energy values for the MB uptake by AC and FeAC (21.79 and 14.82 kJ/mol, respectively) revealed a physisorption process. In the regeneration study, FeAC has shown consistently ≥ 90% MB removal even up to 10 repeated cycles. The reusable characteristic of the spent FeAC improved the practical use of activated carbon and can be a breakthrough for continuous flow system applications where it can work effectively without any significant reduction in its performance.

## Introduction

The provision of fresh air and clean water are very essential to living organisms. Hence, the preservation of the quality of fresh water resources remains the all times challenges. Certainly, the sludge and residues released from industrial sites contain hazardous pollutants, which deteriorate the water quality [1]. The presence of toxins such as metals, dyes, pesticides and many antibiotics released as effluents from the industrial operations and agricultural runoff is creating serious threats to the environment. Synthetic dyes impart color to the wastewaters which ultimately disturb the growth activity of aquatic organisms and are responsible for the increase in chemical oxygen demand (COD) [2]. Moreover, dyes inhibit the photosynthesis process by absorbing the sunlight, which is necessary for aquatic plants growth. These pollutants are also threatening the food cycle and aquatic life due to their mutagenic character and carcinogenicity [3]. The health risks associated with dyes are cyanosis, vomiting, jaundice, accelerated heart rate, shock and tissue necrosis. It is therefore vital to treat the industrial effluents containing dyes before they enter the water system. Numerous physico–chemical methods, such as coagulation and oxidation [4], adsorption [5], nano–filtration and flocculation [6], ion exchange [7] and membrane process [8] have been used to remove the synthetic dyes from the wastewater and industrial effluents. However, adsorption has been found to be the most suitable technique due to its low cost, accuracy, viability and simple design requirements [9,10].

Activated carbon (AC) has been widely used as an adsorbent in the purification of aqueous media, gas/solid phase separation, catalysis, electrochemical processes etc. The surface characteristics of activated carbon, i.e. the extended range of porosity and high surface area, ease of separation, low operational cost and significant sorption affinity make AC a versatile and preferred material for various applications [11,12]. To further improve its efficiency, research on the modification and the reusability of AC has been carried out [10].

This paper elaborates on the surface modification of AC to improve its sorption affinity towards MB and the regeneration of the spent adsorbent. In order to generate more active sites on AC, the surface was modified by using an oxidizing agent followed by the impregnation with an iron precursor to produce iron impregnated AC (FeAC). Both the AC and FeAC were thoroughly characterized and investigated for the MB uptake from aqueous systems. For practical applications, the reusability of the spent adsorbent is of prime economic interest, firstly to reduce operating cost and secondly to solve the problems related to the disposal of the spent adsorbent which is a costly option and a waste of resources. This paper reports the enhanced MB removal efficiency by iron impregnated AC under different experimental conditions and the reusability of the spent adsorbents in repeated cycles.

## Materials and Methods

### Materials

Analytical grade reagents were used as received without further purification. AC was purchased from R & M Chemicals, Essex (UK). Sodium bicarbonate (NaHCO_3_) and ferrous sulphate (FeSO_4_.7H_2_O) were provided by ACROS Organics, USA. Methylene Blue and sodium nitrate (NaNO_3_) were obtained from QReC (New Zealand) while potassium permanganate (KMnO_4_) was received from Amresco (USA).

### Synthesis of FeAC

The as received AC was heated for 2 h at 110°C in an oven to ensure moisture free substrate. The synthesis of FeAC was carried out by following the procedure described in our recently reported work [13]. Initially, 5 g AC was stirred with 1 M KMnO_4_ solution for 20 min at 200 rpm. Afterwards, distilled water was added to dilute the suspension before it was filtered. The residue obtained was then mixed with 1 M FeSO_4_.7H_2_O and stirred on a magnetic stirrer (WiseStir, DAIHAN Scientific Co. Ltd.), up to 8 h at the same speed. The suspension was filtered, washed with 1% NaHCO_3_ and soaked in 1% NaHCO_3_ solution overnight. Later, the suspension was decanted, washed with distilled water and filtered again. Finally, the solid residue was air dried for 2 h and then kept in an oven at 110°C up to 6 h for complete drying.

### Characterizations of the adsorbents

The pH of zero point of charge (pH_*ZPC*_) of AC and FeAC was determined following the well known salt addition method reported in literature [14]. To a series of Erlenmeyer flasks (100 mL), 40 mL of 0.1 M NaNO_3_ solution was added and the pH was adjusted by using HNO_3_ and NaOH with variable concentrations (0.1–1 M) in the pH range of 2–10. To each flask, 0.1 g adsorbent was added and the suspensions were agitated at a stirring speed of 250 rpm overnight at ambient temperature. The next day, final pH (pH_*f*_) of the suspensions was recorded and the difference between final and initial pH (ΔpH) was plotted against initial pH (pH_*i*_). The pH value where net surface charge was zero, is considered to be the zero point of charge (pH_*ZPC*_) of the material. A small quantity of the adsorbents was used to investigate the presence of surface functional groups by using FTIR spectroscopic technique (Model Perkin Elmer Spotlight 200) equipped with a mercury cadmium telluride (MCT) detector.

### Sorption studies of Methylene Blue (MB)

In order to investigate the MB removal efficiency by AC and FeAC, MB sorption studies were conducted under different experimental conditions and parameters including variable pH, time of contact at various temperatures (298–328 K), adsorbent dosage and ionic strength of the background electrolyte. To examine the effect of solution pH on the uptake of MB by AC and FeAC, 40 mL aliquot (500 mg/L MB) was transferred into a series of 100 mL Erlenmeyer flasks. To each solution, 0.1 g adsorbent was added and allowed to mix for 3 h at 400 rpm using an orbital shaker Model IKA, KS 260 basic. Each time, the pH adjustment was made by using HNO_3_ or NaOH (0.1–1 M) in the pH range of 2–10. After equilibration, the suspensions were filtered and the remaining MB concentration in the filtrate was determined at λ = 660 nm using a Shimadzu UV-VIS 2600 spectrophotometer Version 1.03, equipped with the UV Probe 2.42 software.

To evaluate the effect of contact time at different temperatures (298–328 K) on MB removal by AC and FeAC, 0.1 g solid adsorbent was added to a series of Erlenmeyer flasks (100 mL). To each flask, a 100 mg/L MB solution (40 mL) was introduced and the suspension was stirred on a magnetic stirrer for pre–defined time intervals at various temperatures (298, 313 and 328 K) for up to 4 h. Similarly, the effect of adsorbent dosage on the MB removal from aqueous solutions was investigated by using various adsorbent doses (0.1–1 g). In a series of Erlenmeyer flasks (100 mL), 40 mL of MB solution (500 mg/L) was mixed with the pre-weighed adsorbents. The suspensions were agitated at 400 rpm for 3 h and the remaining MB concentration was determined in the same manner.

During the release of wastewater, the discharge may also contain accompanying ions such as Na^+^, K^+^ etc. In order to understand the effect of ionic strength on MB degradation rate or process, different solutions of background electrolyte (0.01–1 M NaNO_3_) and a blank (distilled water) with MB solution were analyzed. These studies were conducted at the natural pH of the suspensions containing 0.1 g adsorbent, 20 mL MB solution (1 to 70 mg/L) and/or 20 mL distilled water/ NaNO_3_ solution which were allowed to mix for 5 h at 250 rpm using an orbital shaker Model IKA Basic 260. In all the cases, the amount of MB adsorbed, *q*_*e*_ (mg/g) on the solid adsorbent was determined by using Eq 1,

$$q_{e}=\frac{(C_{o}-C_{e})V}{m}$$ (1)

where *C*_*o*_ and *C*_*e*_ (mg/L) are the initial and equilibrium concentrations of MB, *V* (mL) is the volume of MB used and *m* (g) is the mass of solid adsorbent. The MB removal efficiency of AC and FeAC was computed by using Eq 2,

$$\text{Removal}\;\left(\%\right)=\frac{(C_{o}-C_{e})100}{C_{o}}$$ (2)

The economic feasibility of the adsorption process is dealt with the regeneration of the spent adsorbents in practical applications. The MB loaded AC and FeAC were eluted by using different desorbing agents (0.1 M H_2_SO_4_, 0.1 M NaOH, 0.1 M NaNO_3_ and distilled water) to evaluate the reusability of the MB loaded adsorbents. In general, 1 g of adsorbent was stirred with 50 mg/L MB solution for 3 h using an orbital shaker. After equilibration, the MB concentration in the analyte was determined. The MB loaded AC and FeAC were agitated with the eluents for the same duration as that of the reaction time (3 h). The amount of MB desorbed (% desorption) in each case was computed by using the following relationship;

$$\text{Desorption}\;(\%)=\frac{C_{des}}{C_{ads}}\times 100$$ (3)

where *C*_*des*_ and *C*_*ads*_ (mg/L) represent the concentration of dye in desorbed and adsorbed phases, respectively. After each treatment, the adsorbent material was separated from the dye solution and washed with distilled water. Finally, it was soaked in distilled water overnight. After filtration, the solid residue was again agitated with the fresh 40 mL MB solution (50 mg/L) for another cycle. The process was repeated for up to 10 cycles and the MB removal efficiency was computed after each cycle.

## Results and Discussion

### Characterization of AC and FeAC

**Fig 1:**
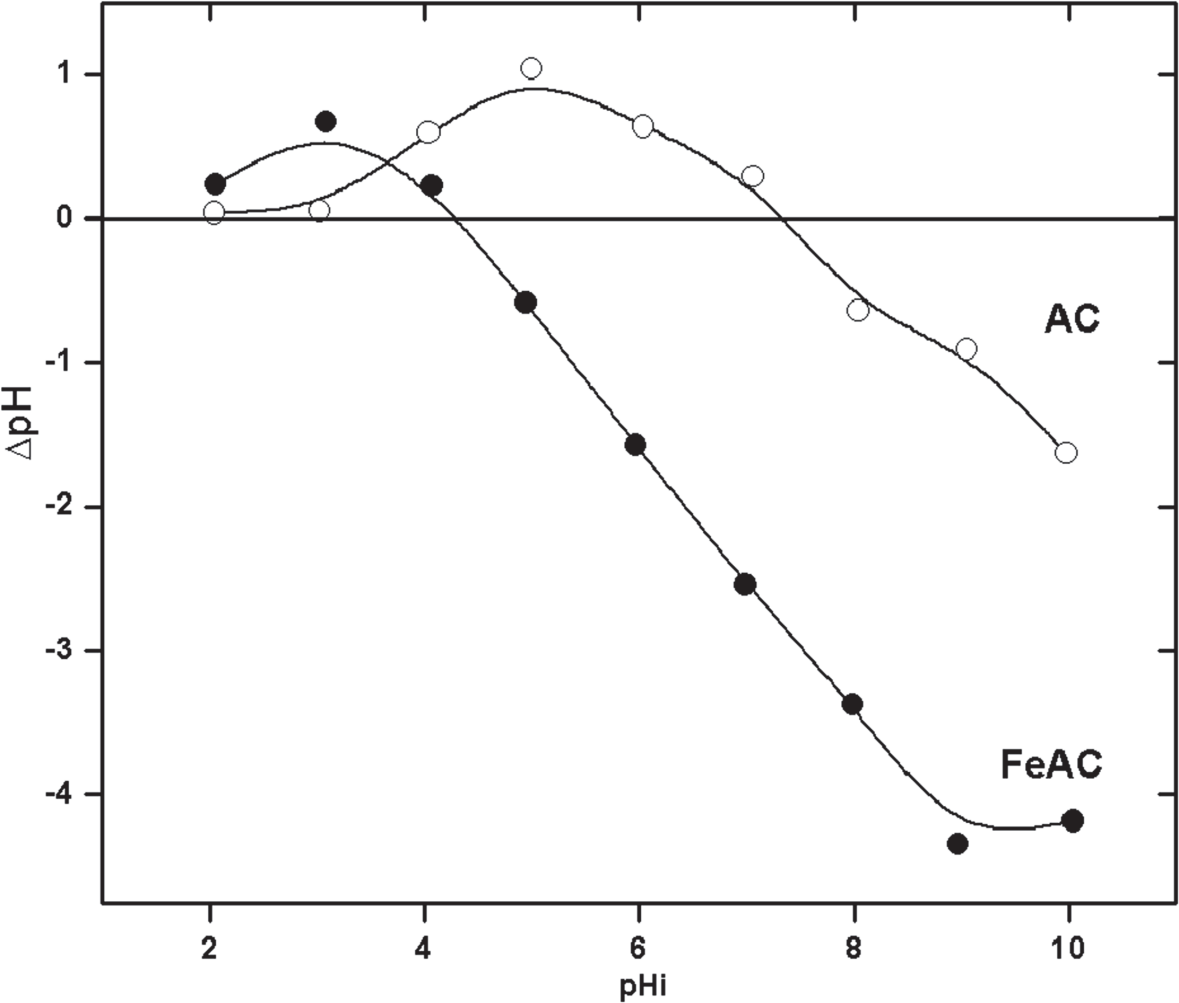
Determination of the pH of zero point of charge (pH_*ZPC*_) for AC and FeAC.

The pH of zero point of charge (pH_*ZPC*_) corresponds to the pH value at which the surface of the solid is considered to be neutral. It plays an important role during the sorption of ionic species on solid surfaces from aqueous systems. The plots of pH_*ZPC*_ for both the AC and FeAC are depicted in Fig 1.

The oxidation followed by the iron impregnation of the carbon surface has changed the pH of the modified carbon sample. The results indicate that the surface of FeAC is more acidic (pH_*ZPC*_ = 4.3) whereas the surface of AC is close to neutrality (pH_*ZPC*_ = 7.4). The lower pH_*ZPC*_ value renders FeAC to be more suitable in the removal of cationic dyes such as MB from contaminated water systems. The increased in acidic character of FeAC reveals that the degree of protonation is inevitably affected by iron impregnation. The modification further contributes to the generation of weak acidic functional groups such as phenolic, lactonic, carbonyl and carboxylic groups on the surface. At pH values below pH_*ZPC*_, the FeAC surface is positively charged while at pH above pH_*ZPC*_ the surface of FeAC is negatively charged. At lower pH values (pH 2 and 3) the surface of FeAC undergoes surface protonation due to the frequent interaction and accumulation of H^+^ ions from the bulk, which have the tendency to surround the surface of the adsorbent. Besides that, the FeAC surface is assumed to release the basic OH^−^ ions into the bulk which results in a slight increase in the final pH of the suspension. Hence, this results in an overall positively charged FeAC surface at lower pH values (below pH_*ZPC*_). However, at alkaline pH (or above the pH_*ZPC*_ of FeAC), the surface of FeAC was found to be negatively charged. Generally, with the increase in initial pH of the system, the excessive amount of OH^−^ ions in the bulk solution are counter balanced by the H^+^ ions liberated from the FeAC surface. Moreover, the deprotonation of FeAC results in the decrease in the final pH of the suspension and creates an overall negative charge on the FeAC surface. The trend in pH_*ZPC*_ values obtained for both the AC and FeAC in this work is in close agreement with the reported literature [15,16]. Previously, Lu et al. [15] observed a decrease in the pH_*ZPC*_ of oxidized carbon samples modified with nitric acid, from 7.72 (AC parent material) to 2.01 for the oxidized AC (v/v ratio of HNO_3_ to water, 5:10). The fairly low pH_*ZPC*_ values of these activated carbons were supposed to be due to the oxidation as well as the increase in acidic surface functional groups on the carbon surface. In another study, the pH_*ZPC*_ values of commercial activated carbon and H_2_SO_4_ modified carbon black were found to be 6.4 and 3.5, respectively [16]. Herein, the significant decrease in the pH_*ZPC*_ values from 7.4 (AC) to 4.3 (FeAC) is attributable to the oxidation as well as the anchoring of iron (Fe) contents on the AC surface upon Fe impregnation. The physicochemical characteristics of AC and FeAC are summarized in Table 1.

**Table 1:** Physicochemical characteristics of AC and FeAC.

Parameter	Adsorbent	
	AC	FeAC
Density (g/cm^3^)	0.41	0.55
pH	7.76	4.93
pH_*ZPC*_	7.4	4.3
Surface area (m^2^/g)	1094	543
Pore width (Ǻ)	17.23	22.98
Total pore volume (cm^3^/g)	0.47	0.31

**Fig 2:**
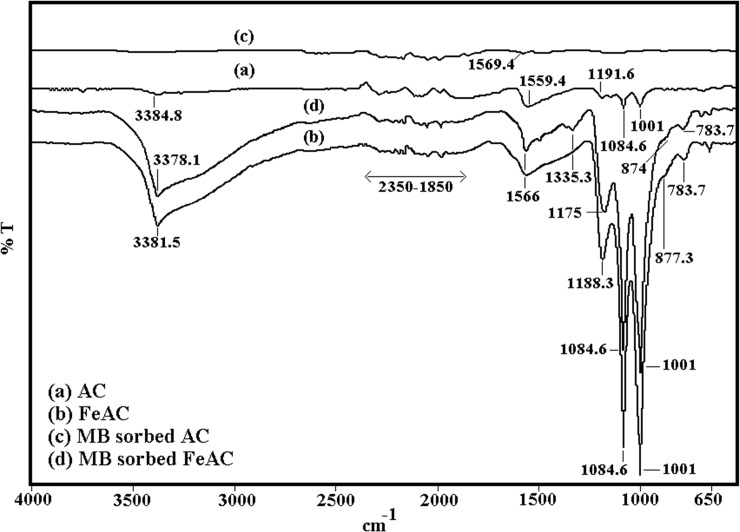
FTIR spectra of AC and FeAC before (a,b) and after MB adsorption (c,d) respectively.

The FTIR spectra for both samples (before and after MB adsorption) were recorded in the range of 4000–650 cm^-1^ and is depicted in Fig 2. The two characteristic absorption peaks appearing at 3384.8 and 3381.5 cm^-1^ in AC and FeAC spectra (before MB adsorption) can be assigned to the moisture contents/chemisorbed water in the sample matrix or due to the O–H stretching and bending vibrations as suggested by Liu et al. [17]. However, in the MB sorbed AC this peak was not detected, while the same peak was slightly shifted to 3378.1 cm^-1^ in FeAC (after MB adsorption). After MB adsorption on FeAC, a weak absorption band at 1335.3 cm^-1^ which corresponds to the C = C of aromatic ring [18] was also observed. Before MB adsorption, a small peak at 1191.3 cm^-1^ in AC but comparatively sharper peak at 1188.3 cm^-1^ in FeAC due to the stretching vibration of C–OH [19] was detected. This peak was then slightly shifted to 1175 cm^-1^ in FeAC after MB sorption. Two peaks found at 877.3 and 783.7 cm^-1^ in FeAC before MB sorption was slightly shifted to 874 cm^-1^ after MB adsorption can be ascribed by the metal–oxygen interactions i.e., α–FeOOH and Fe–OH, as described by Li et al. [20] in a separate study. However, no such peak was found in the FTIR spectrum of AC.

### Adsorption studies

#### Effect of pH on Methylene Blue removal

**Fig 3:**
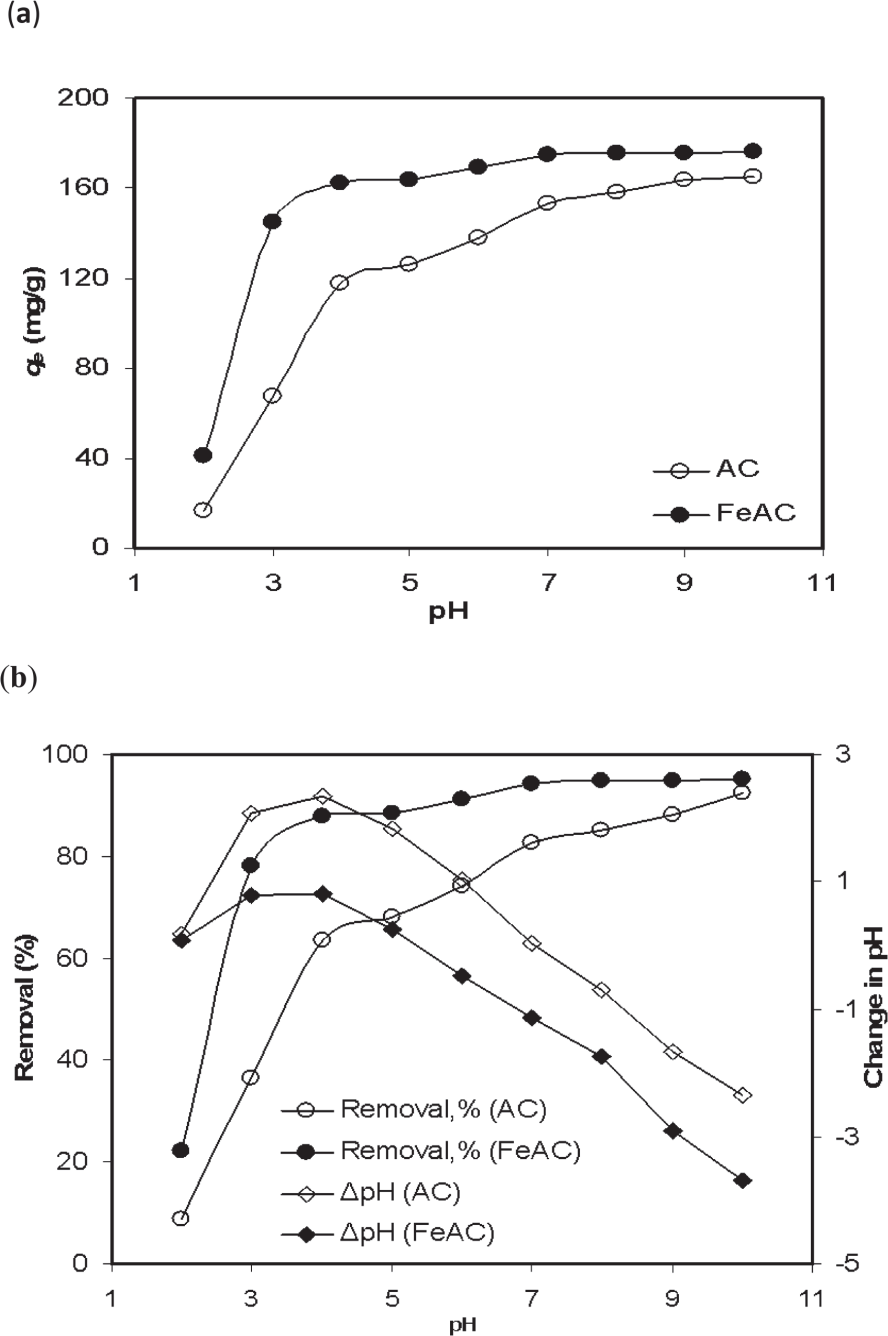
MB uptake by AC and FeAC (a) at different pH (b) represents removal efficiency and pH changes.

The pH of a system is an important parameter used to evaluate the adsorption capacity during the adsorption process as it can influence the dissociation of functional groups available on the adsorbent’s surface, the surface charge density of the adsorbent and its structure. It can also affect the degree of ionization of the adsorbate molecules and is accountable for the higher/lower sorption capacity of the adsorbent [21]. Generally, the trend in dye removal efficiency by an adsorbent can be explained on the basis of the zero point of charge (pH_*ZPC*_) of the adsorbents and the nature of the dye molecules (either anionic or cationic). In the present case, the MB sorption on AC and FeAC was examined in the pH range of 2–10 and the results are shown in Fig 3A.

As can be seen from Fig 3A, pH has a positive effect on the uptake of MB molecules from the aqueous system by both AC and FeAC. The MB removal efficiency by AC was increased from 8 to 89% upon changing the pH from 2 to 10, respectively. Meanwhile, the MB removal efficiency of FeAC was also found to increase from 22% (at pH 2) to 95% (at pH 10). This substantial increase in the MB removal efficiency of AC and FeAC can be well described on the basis of pH_*ZPC*_. As mentioned earlier, below pH_*ZPC*_, the surface of FeAC is positively charged which results in a competition between the H^+^ ions and dye cations to reach the surface. Eventually, the active sites on the FeAC surface will be surrounded mostly by the H^+^ ions and to some extent by the dye molecules. The H^+^ ions will limit the interaction between the cationic MB molecules (MB^+^) and the surface of FeAC. These repulsive forces hinder the contact of positively charged MB molecules to the surface of FeAC and contribute to the small amount of dye adsorbed on FeAC at pH < 4 (140 mg/g). However, a sudden increase in the amount of MB adsorbed on FeAC was noticed at pH > 4 (up to 174.9 mg/g). The maximum amount adsorbed remains almost unchanged in the pH range of 7–10 as there was no significant change in the amount of MB adsorbed on FeAC. Likewise, AC followed a similar trend in the MB uptake below and above its pH_*ZPC*_ value 7.4, but the overall MB adsorption capacity of AC was significantly lower than FeAC at lower pH values. Although the sorption capacity of AC increases at pH ≥7, it is still smaller as compared to that of FeAC (i.e., 153.58 vs. 174.9 mg/g at pH 7, while 165.3 vs. 176.37 mg/g at pH 10, respectively).

The uptake of MB cations increases with the increase in pH and this trend is more dominant above the pH_*ZPC*_ values for both the AC and FeAC, which ultimately approaches the state of equilibrium around pH 9 and 7, respectively. It is pertinent to mention that, the increase in amount of MB adsorbed at pH > pH_*ZPC*_ in both the systems (AC and FeAC) is attributed to the increase in the attractive forces between the negatively charged surface (above pH_*ZPC*_) of AC and FeAC, and the positively charged MB molecules. At alkaline pH there is relatively no competition between the H^+^ ions and MB cations to be adsorbed on the surface of the solid.

The cation exchange capacity (CEC) of adsorbent to the bulk media can be explained as follows; at higher pH values (above pH_*ZPC*_), the H^+^ from the surface of adsorbent were liberated and moved to the bulk. This is also evidenced from the decrease in the final pH values of each solution (above pH_*ZPC*_) which can correspondingly increase the percentage of MB removal, as shown in Fig 3B. Due to deprotonation from FeAC surface, the positively charged dye molecules are expected to be attracted by the FeAC surface. This results in the high MB adsorption capacity of the acidic FeAC. Hence, it can be stated that both the electrostatic forces and CEC are responsible for the significant uptake of dye molecules by FeAC at higher pH values. It is also worth mentioning that the MB removal capacity of FeAC is significantly higher (176.37 mg/g at pH 10) than other sorbent materials such as graphite (< 50 mg/g) and oxidized graphite (<150 mg/g) [22] and hydrogen–titanate nanofibres (16.69 mg/g at pH 9) [23] previously used for the removal of MB at different pH values.

#### Adsorption kinetics studies

The MB adsorption kinetics and the dye sorption mechanism on AC and FeAC surfaces at different temperatures were also evaluated. To evaluate the data obtained, different kinetics models (i.e., pseudo–first order, pseudo–second order, intraparticle diffusion, Bangham model and Elovich equation) were used.

#### Pseudo–first order model

**Fig 4:**
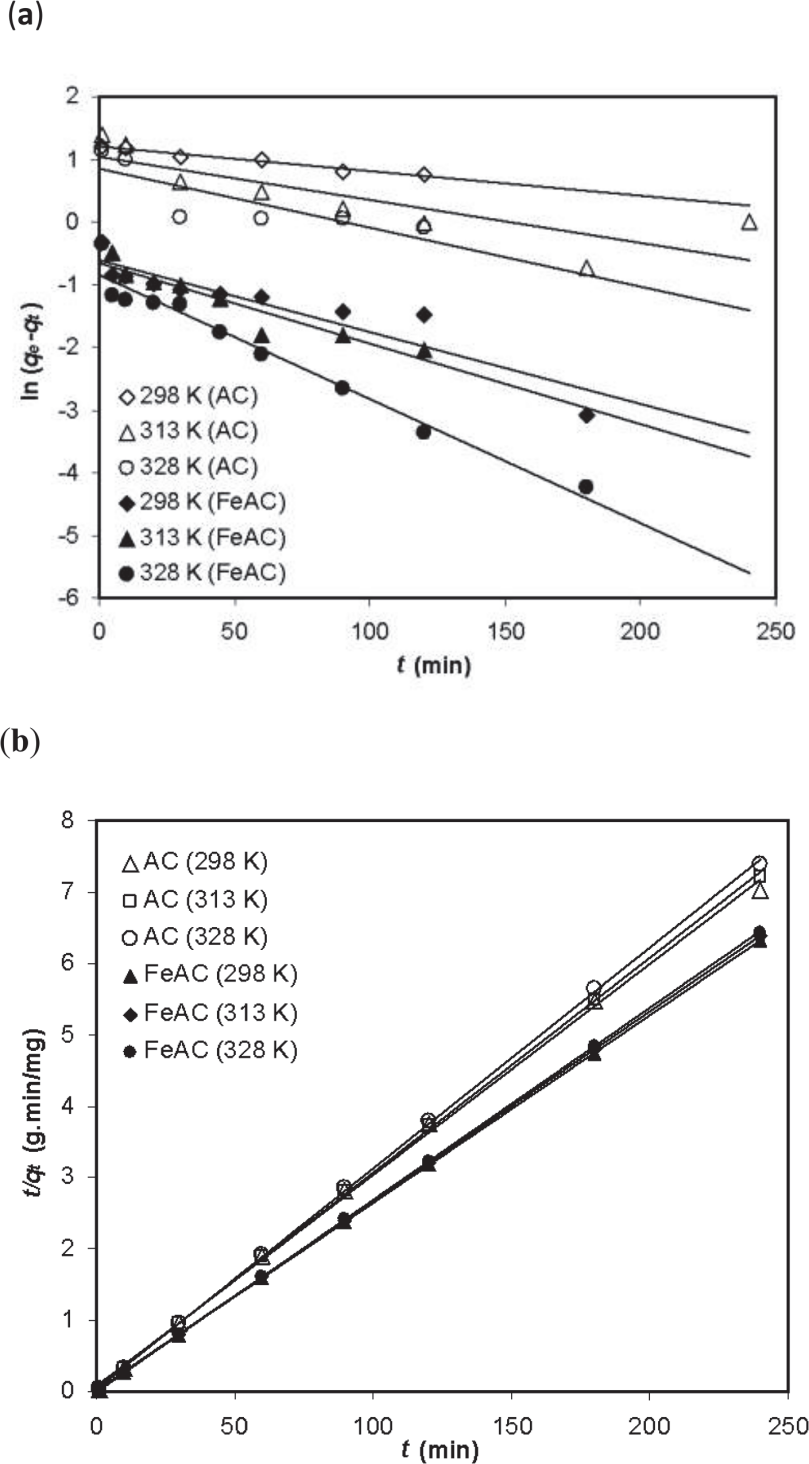
Kinetic investigations on AC and FeAC (a) pseudo–first order (b) pseudo–second order models.

This model deals with the solid adsorption capacity of the adsorbent towards adsorbate molecules and is given in its linear form as;

$$\text{ln}(q_{e}-q_{t})=\text{ln}\;q_{e}-k_{1}t$$ (4)

where *q*_*e*_ (mg/g) is the amount of the solute adsorbed per unit weight of the adsorbent at equilibrium and *q*_*t*_ (mg/g) is the amount adsorbed at time *t* (min), while *k*_*1*_ (1/min) is the rate constant for pseudo–first order rate expression. The plots between ln (*q*_*e—*_ *q*_*t*_) versus *t* at various temperatures is shown in Fig 4A.

The slope and intercept derived from the plots of ln (*q*_*e—*_ *q*_*t*_) versus *t* were used to calculate the values of *k*_*1*_ and *q*_*e*_, and the values are given in Table 2. where, *q*_*e*_,_exp_, *q*_*e1*,cal_ and *q*_*e2*,cal_ (mg/g) represents the amount adsorbed at equilibrium experimentally, and according to pseudo–first order and pseudo–second order models, respectively. The low values of correlation coefficient (*r* ^2^) and the large difference between experimental and calculated values of the maximum amount adsorbed (see Table 2) indicates that pseudo–first order model is not suitable to explain the reaction mechanism for the dye uptake by both the adsorbents (AC and FeAC).

**Table 2:** Kinetic parameters for the MB sorption by AC and FeAC at various temperatures.

**Adsorbent**	**Parameter**	**Temperature (K)**		
		**298**	**313**	**328**
	*q*_*e*_,_exp_ (mg/g)	34.19	33.22	32.49
**AC**	**Pseudo–first order rate model**			
	*q*_*e1*_,_cal_ (mg/g)	3.289	2.783	2.353
	*k*_*1*_ *×*10^3^ (1/min)	3.9	6.9	9.5
	*r* ^2^	0.970	0.701	0.674
	**Pseudo–second order rate model**			
	*q*_*e2*_,_cal_ (mg/g)	33.89	33.20	32.36
	*k*_*2*_ *×*10^3^ (g/mg min)	9.93	15.46	22.2
	*h* (mg/g min)	11.41	17.06	23.25
	*r* ^2^	0.998	0.999	0.999
	**Intraparticle diffusion model**			
	*k*_*i*_ (mg/g min^0.5^)	0.373	0.205	0.134
	*I*	28.18	30.01	30.23
	*r* ^2^	0.931	0.996	0.873
	**Bangham kinetic model**			
	*k*_*r*_ (mg/g min)	30.26	28.81	28.61
	*m*	71.43	43.47	47.61
	*r* ^2^	0.609	0.943	0.854
	**Elovich equation**			
	*R*_*E*_	0.087	0.07	0.035
	*r* ^2^	0.924	0.945	0.968
**FeAC**	**Parameter**	**Temperature (K)**		
		**298**	**313**	**328**
	*q*_*e*_,_exp_ (mg/g)	38.01	37.57	37.25
	**Pseudo–first order rate model**			
	*q*_*e1*_,_cal_ (mg/g)	0.547	0.512	0.43
	*k*_*1*_ *×*10^3^ (1/min)	11.5	12.8	19.8
	*r* ^2^	0.863	0.895	0.964
	**Pseudo–second order rate model**			
	*q*_*e2*_,_cal_ (mg/g)	38.02	37.59	37.31
	*k*_*2*_ *×*10^2^ (g/mg min)	133.01	141.51	231.69
	*h* (mg/g min)	192.3	200	322.58
	*r* ^2^	1	0.999	1
	**Intraparticle diffusion model**			
	*k*_*i*_ (mg/g min^0.5^)	0.044	0.03	0.018
	*I*	37.37	37.12	36.99
	*r* ^2^	0.933	0.895	0.883
	**Bangham kinetic model**			
	*k*_*r*_ (mg/g min)	37.37	36.74	36.63
	*m*	500	250	333
	*r* ^2^	0.83	0.968	0.927
	**Elovich equation**			
	*R*_*E*_	0.013	0.015	0.012
	*r* ^2^	0.783	0.782	0.547

#### Pseudo–second order model

The data obtained at various contact time between the solute molecules to the adsorbents at various temperatures (298, 313 and 328 K) were also analyzed using the pseudo–second order model shown in the linear form as;

$$\frac{t}{q_{t}}=\frac{t}{q_{e}}+\frac{1}{k_{2}q_{e}^{\;2}}$$ (5)

where *q*_*e*_, *q*_*t*_ and *t* represent the same variables as mentioned above while *k*_*2*_ (g/mg min) is the rate constant for the pseudo–second order rate expression for the solid phase adsorption. The plots of *t/q*_*t*_ versus *t* showing the contact time effect on dye uptake by AC and FeAC at different temperatures (298, 313 and 328 K), are given in Fig 4B. The values of rate constants (*k*_*2*_) and the amount adsorbed at equilibrium (*q*_*e*_) have been determined from the slopes and intercepts of the linear plots, respectively and is shown in Table 2. A slight decrease in the sorbed amount of MB on FeAC with the increase in temperature was observed (38.05, 37.57 and 37.25 mg/g at 298, 313 and 328 K, respectively). AC also shows the same trend towards MB sorption (33.89, 33.2 and 32.36 mg/g at 298, 313 and 328 K, respectively). The amount of MB adsorbed (38.05 mg/g) on FeAC is almost 3 times higher than the amount of MB sorbed (12.66 mg/g) on mesoporous aluminophosphate (AlPO_4_) observed by Kannan et al. [24]. The initial rate of sorption *h* (mg/g min) was also computed by using the relation as follows,

$$h=k_{2}q_{e}^{\;2}$$ (6)

The values of ‘*h*’ (Table 2) for MB sorption by FeAC are much higher (≥10 fold) than its counterpart AC, which indicates a faster removal rate of MB by FeAC compared to AC. The activation energy, *E*_*a*_ (kJ/mol), for the adsorption processes by the two adsorbents was determined by using the linear form of Arrhenius equation;

$$\text{ln}\;k_{2}=\text{ln}\;A-\frac{E_{a}}{RT}$$ (7)

where *k*_*2*_ is the pseudo–second order rate constant, *A* (g/mg min) is the pre–exponential Arrhenius factor, *R* (8.3145 J/mol K) is the gas constant and *T* (K) is the absolute temperature. From the slopes of linear plots of ln *k*_*2*_ versus 1/*T* (not shown), the values of activation energies were calculated. The low values of activation energies (21.79 and 14.82 kJ/mol for both the AC and FeAC, respectively) refer to the low energy barrier for the reactions studied as well as the physisorption process [25]. The activation energy values for both the systems are also pointing towards diffusion controlled sorption processes as suggested by Sen et al. [26]. The values of *r* ^2^ > 0.99 and the close agreement between the experimental and calculated values of the maximum amount of MB adsorbed at corresponding temperatures (Table 2), indicates that the pseudo–second order model is the best model to describe the data analyzed for both the AC and FeAC systems. In addition, three other kinetics models were also tested to understand the reaction mechanism and the overall rate of the adsorption process.

#### Intraparticle diffusion model

Generally, the dye removal from aqueous systems through an adsorption process follows the following steps [27]; (1) dye molecules migrate from the bulk to the surface of sorbent material via bulk diffusion, (2) dye molecules diffuse through the surface boundary layer of adsorbent via film diffusion, (3) dye molecules migrate from the exterior of surface to the interior pores of the material via intraparticle diffusion or pore diffusion, and lastly (4) the adsorption of dye molecules on the active sites of the adsorbent’s surface via ion exchange, chelation and/or complexation pathway. However, for dyes with larger molecular size (e.g. MB) adsorption on particular adsorbent is a diffusion controlled process, either through intraparticle mass transfer, external film resistance or liquid phase migration of the dye molecules. In such case, the rate of diffusive mass transport of the dye molecules can be expressed as [28];

$$q_{t}=k_{i}t^{0.5}+I$$ (8)

where, *q*_*t*_ (mg/g) is the amount of MB adsorbed at time *t* (min), *k*_*i*_ (mg/g min^0.5^) is the rate constant for intraparticle diffusion model and the intercept (*I*) is an indicative of the boundary layer thickness effect. The boundary layer effect on adsorption process is low if the magnitude of *I* is small. Both, *k*_*i*_ and *I* can be obtained from the linear plots of *q*_*t*_ versus *t* ^0.5^ (Figure not shown here). The plots have shown multi-linearity with two different adsorption stages. The first linear portion corresponds to the external mass transfer (i.e. MB molecules migrate towards AC and FeAC surface through film diffusion), with a faster rate. Whereas, the second linear portion is correlated to the gradual uptake of MB by AC and FeAC through intraparticle diffusion with rate constants, 0.373, 0.205 and 0.134 mg/(g min^0.5^) for AC, while 0.044, 0.03 and 0.018 mg/(g min^0.5^) for FeAC at 298, 313 and 328 K, respectively. Since the straight lines did not pass through the origin, this confirmed that the film diffusion as well as intraparticle diffusion occurred simultaneously during the MB sorption on AC and FeAC [27]. As for any adsorption process, if *I* ≠ 0, the reaction process is quite complex and is not solely controlled by the intraparticle diffusion, as suggested by Wang et al. [29]. The details of *k*_*i*_, *I* and *r* ^2^ given in Table 2, infer that intraparticle diffusion is one of the rate controlling steps during the MB sorption by AC and FeAC.

#### Bangham kinetic model

To illustrate the role of pore diffusivity for the current case, Bangham kinetic model proposed by Aharoni et al. [30] was used.

$$q_{t}=k_{r}t^{1/m}$$ (9)

where, *q*_*t*_ (mg/g) is the amount of MB adsorbed at any time *t* (min), *k*_*r*_ (mg/g min) is the rate constant for MB sorption and 1/*m* indicates the adsorption intensity. The values of *k*_*r*_ and *m* (Table 2) have been calculated based on the linear plots between ln*q*_*t*_ vs. ln*t* (not shown). Although some of the *r* ^2^ values were less than 0.9, but the *k*_*r*_ values for AC (30.26, 28.811 and 28.61 mg/g min) were close to the amount adsorbed (*q*_*t*_) at respective temperatures (Table 2). Similarly, the *k*_*r*_ values for FeAC (37.37, 36.74 and 36.74 mg/g min) correlate well with the *q*_*t*_ (mg/g) at all temperatures. Meanwhile, the linearity of the straight lines at 313 and 328 K (*r* ^2^ > 0.90) further supports that the pore diffusion mechanism also plays an important role during the MB uptake by both AC and FeAC [31]. However, the non-linearity of the straight lines at 298 K (*r* ^2^ < 0.90) indicates the deviation from the Bangham model. Thus, it can be inferred that pore diffusion model cannot control the overall rate of the reaction but is applicable along with the intraparticle diffusion and film diffusion [32].

#### Elovich equation

This equation is used to signify the decreasing rate during adsorption process with the passage of time, due to the increase in surface coverage. The following linear form of Elovich equation was applied, as suggested by Chien and Clayton [33];

$$q_{t}=\frac{1}{\beta }\text{ln}(\alpha .\beta )+\frac{1}{\beta }\text{ln}\;t$$ (10)

where *q*_*t*_ has the same meanings as discussed earlier, *α* (mg/g min) is the initial rate of adsorption and *β* is the desorption constant during the adsorption process. However, the dimensionless Elovich equation can be written as [34],

$$(\frac{q_{t}}{q_{ref}})=(\frac{1}{q_{ref}\beta })\text{ln}(\frac{t}{t_{ref}})+1$$ (11)

where, *t*_*ref*_ is the longest duration for an adsorption process and *q*_*ref*_ be the corresponding amount sorbed at *t*_*ref*_. Another important factor, called the equilibrium approaching factor (*R*_*E*_ = 1/*q*_*ref*_ *β*) can be introduced into Eq 11 which will transform it as follows;

$$(\frac{q_{t}}{q_{ref}})=R_{E}\;\text{ln}(\frac{t}{t_{ref}})+1$$ (12)

**Fig 5:**
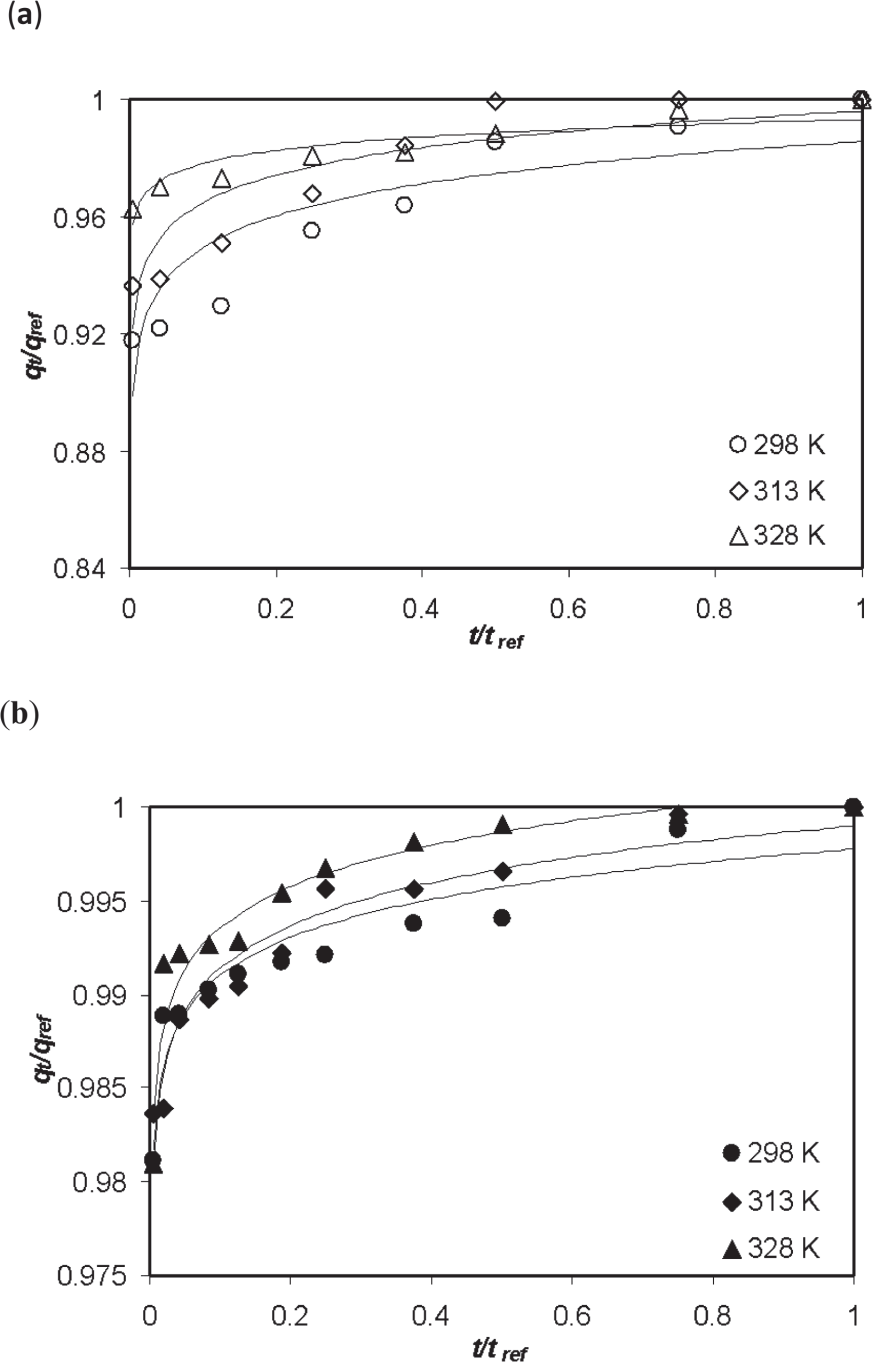
Dimensionless characteristic curves of MB sorption by (a) AC and (b) FeAC at various temperatures.

A plot of *q*_*t*_/*q*_*ref*_ vs *t*/*t*_ref_ (Fig 5), gives information about the *R*_*E*_ values for the MB removal by AC and FeAC at corresponding temperatures.

The curves derived from Eq 12, can be classified into four different zones depending upon the *R*_*E*_ values ranging from 0.01 to 0.3 [34]. In the present case, the *R*_*E*_ values are in the range of 0.1 > *R*_*E*_ > 0.01 (Table 2), suggesting a rapid diffusion of MB molecules from the bulk into the surface of the adsorbent material. In previously reported studies involving the kinetics of MB removal using Elovich equation (Table 3), the *R*_*E*_ values are in the range 0.3 > *R*_*E*_ > 0.1 (zone II) which shows a mild rise in the adsorption curve (moderate diffusion of the pollutants). However, this contradicts with our results as, in our case 0.1 > *R*_*E*_ > 0.02 (zone III), which corresponds to the rapid rise in the adsorption for AC. Meanwhile, the values of *R*_*E*_ < 0.02 (zone IV) for FeAC, corresponds to a quick and approachable equilibrium state.

**Table 3:** Elovich equation’s dimensionless parameter for the kinetics of MB by different types of adsorbents.

Adsorbent	*t*_*ref*_ (min)	*R*_*E*_	Ref.
Pinewood AC	210	0.11	[35]
Apricot AC	60	0.142	[36]
Chitosan bead	270	0.189	[37]
AC (FWKC1060)^a^	30	0.195	[38]
AC (CPKC3)^b^	30	0.199	[39]
AC (CobNa50)^c^	30	0.216	[40]
Activated Carbon (AC)	240	0.087	This work
Iron impregnated activated carbon (FeAC)	240	0.013	This work

Wherein, raw materials for a, b and c were corncob, fir wood and cane pith, respectively.

Among all the kinetics models applied to the experimental data, pseudo-second order model was the best fitted model with the higher *r* ^2^ values (*r* ^2^ > 0.99) and good correlation between the theoretical and experimental *q*_*e*_ values. However, the reaction mechanism was more complex and we found that a number of processes such as film diffusion, pore diffusion and intraparticle diffusion were simultaneously controlling the rate of MB adsorption by AC and FeAC.

### Effect of adsorbent dose

**Fig 6:**
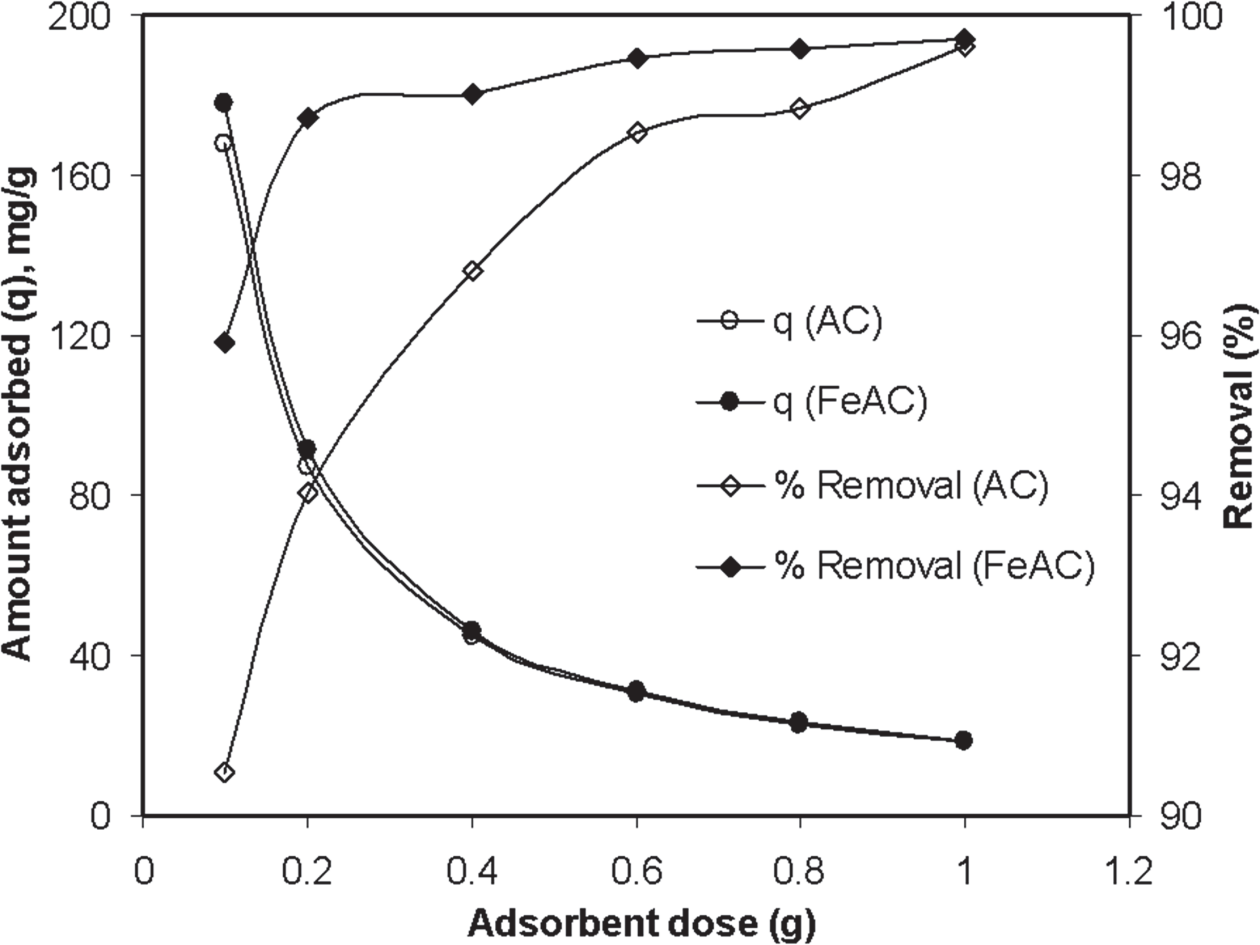
Adsorbent dosage effect on the sorption of Methylene Blue by AC and FeAC.

The MB removal using different adsorbent dosage (0.1–1 g) was also studied and the results for a 500 mg/L MB solution are shown in Fig 6. The MB removal efficiency was observed to increase with the increase in adsorbent dosage. At an initial dose of 0.1 g, both the AC and FeAC have shown 90.5 and 95.9% MB removal efficiency, respectively, which was further enhanced to 99% by both the AC and FeAC when the adsorbent amount was increased to 1 g. As expected, increasing the amount of adsorbent resulted in the increased percent removal for the dye (MB). This can be explained by the fact that, higher dosage of the adsorbent provides more active sites to the adsorbate molecules, which can enhance the toxin removal potential of the adsorbent [41]. The increase in MB uptake by AC and FeAC with the dosage may also be explained by the fact that the solute molecules conglomerates over the surface, provided that some active sites remain unoccupied and available for further adsorption. However, a considerable decrease in the amount of MB sorbed per unit mass of the adsorbent was also observed. The reduction in per unit mass adsorbent can be explained on the basis of the adsorbent to adsorbate ratio i.e., for a fix solute concentration, the adsorption capacity varies depending upon the binding sites available on the surface. For 0.1 g adsorbent, a significant number of MB molecules occupy the available active sites showing higher per unit mass adsorbent value. Whereas for 1 g adsorbent, the number of active sites were increased for the same concentration of MB, hence some of the active sites on the surface remained unoccupied, resulting in a decreased per unit weight adsorbent value.

It is important to highlight, that the MB removal efficiency increased rapidly to 98% with only 0.2 g of FeAC, while AC require higher dosage (more than 0.6 g) to show similar MB removal efficiency. In general, it can be concluded that the overall MB removal efficacy of FeAC was significantly higher (even at low dosage) compared to AC.

### Effect of ionic strength

**Fig 7:**
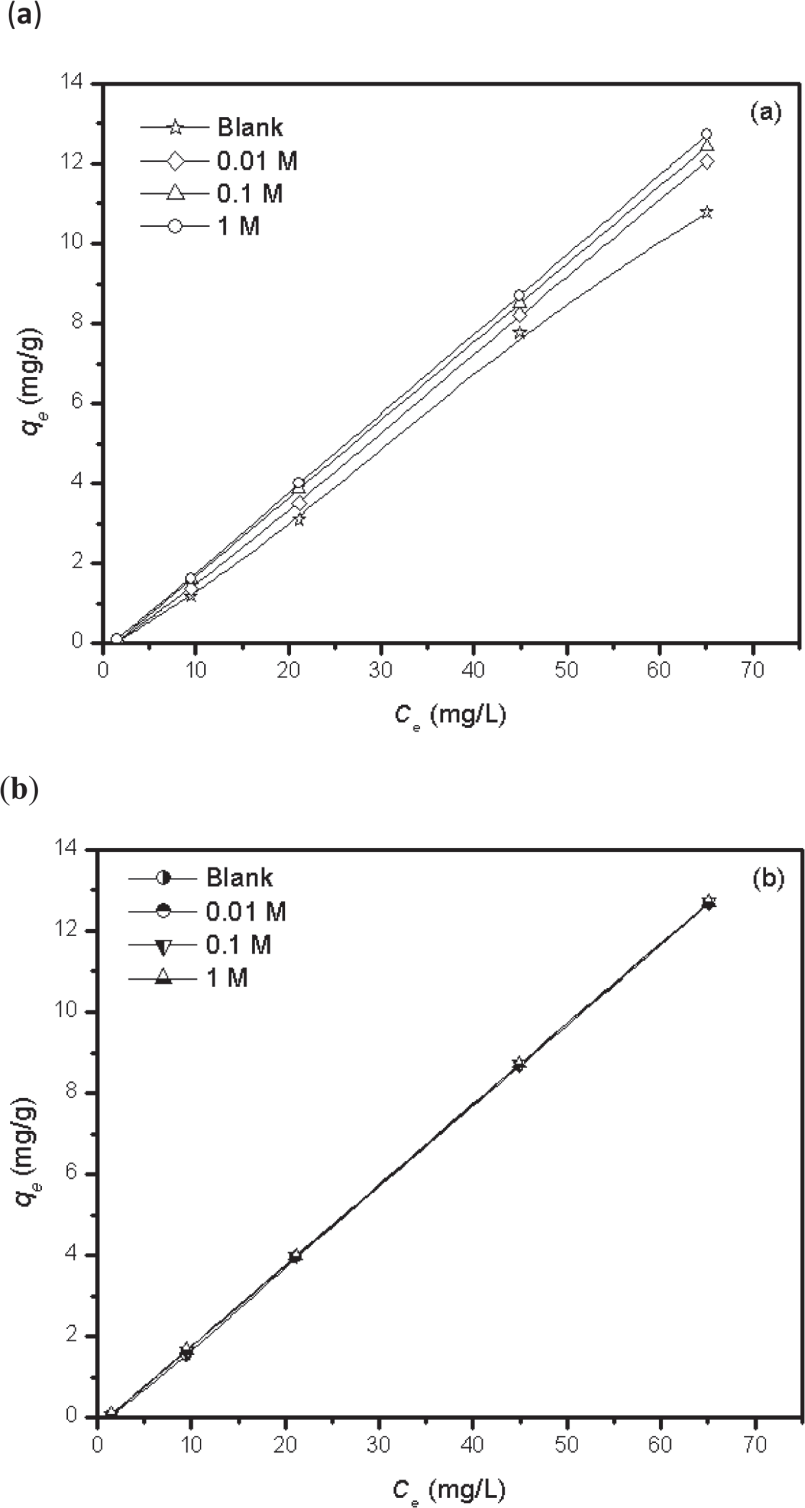
Effect of ionic strength on the MB uptake by (a) AC and (b) FeAC.

It was observed that with the increasing concentration of salt (NaNO_3_) solution from 0.01 to 1 M, the MB removal efficiency by AC was also increased. This can be explained on the basis of pH change (before and after) and the electrostatic interactions following the formation of an electrical double layer (EDL) which turn out to be compressed with the increase in salt concentration [42]. It was observed that for AC, the final pH of each suspension were higher than the initial pH of the solution. This can be inferred as either AC is liberating some OH ions into the bulk or the negatively charged surface functional groups surrounds the H_3_O^+^ ions migrating from the bulk. The similarly charged ions from the electrolyte (i.e., Na^+^) shield the electrostatic repulsion between the paired cationic molecules (from salt and MB^+^ simultaneously) and hinder the solubility of cationic dye molecules into the solvent. Hence, the hydrophobic MB molecules find their way to the surface of adsorbent at a higher salt concentration. This results in the increased amount of MB adsorbed on AC (Fig 7A) which is normally referred to as the salting out effect. Similar results have been reported previously by Benaissa [43] who worked on almond peel for the removal of MB from aqueous solutions.

In contrast, there were no drastic change except for a slightly decreasing trend in the amount of MB adsorbed with the increasing salt concentration (i.e., 0.01–1 M) using FeAC (Fig 7B). Interestingly, the final pH of all suspensions in this case was lower than the initial pH of each system, which could be explained to be due to the deprotonation of the FeAC surface. The positively charged ions from the electrolyte compete with the cationic dye molecules to surround the available active sites on FeAC. At higher salt concentrations, more positive ions from the salts are expected to occupy the available sites on FeAC surface, hence limiting the dye adsorption on FeAC surface. Therefore, it can be concluded that under the studied experimental conditions, increasing the concentration of background electrolyte does not increase MB adsorption by FeAC. A similar explanation for the effect of ionic strength on MB adsorption has also been reported elsewhere [44,45].

### Regeneration of the spent adsorbents

From the economic point of view, the paramount characteristics of an efficient adsorbent are its reusability with significant adsorption capacity and the restoration of its original characteristics upon reuse. The regeneration of the active sites on the spent adsorbent in repeated use is proportional to its stability, which is crucial for the industrial and large scale practical applications. This will reduce the operational cost of the process as well as minimize the ecological risk factor (secondary pollution). Thus an efficient adsorbent must show excellent performance in the adsorption as well as in desorption processes. For such adsorption/desorption analyses, researchers have used various kinds of eluents e.g., acidic, alkaline and deionized water [46–48].

**Fig 8:**
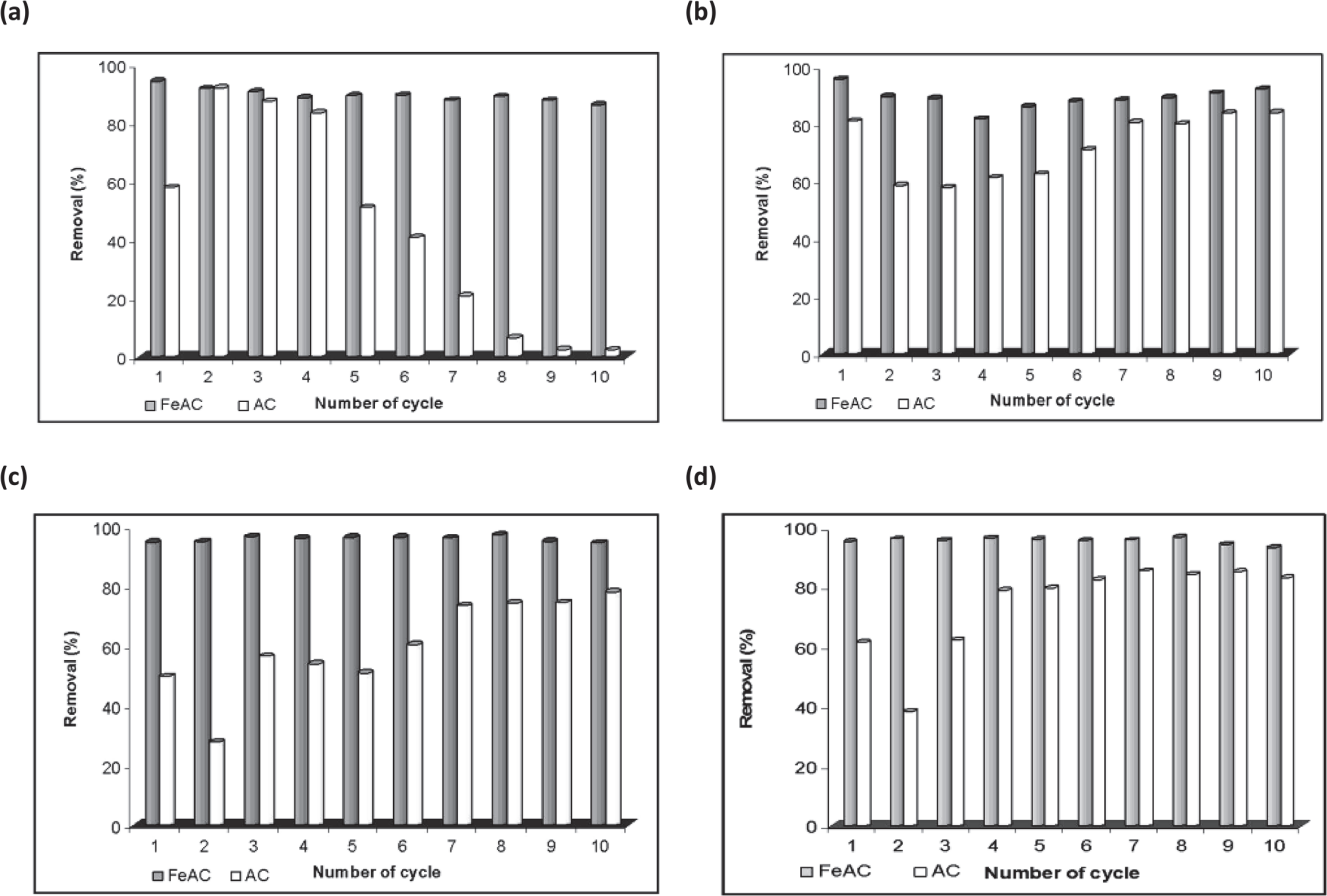
Regeneration studies of spent adsorbents using (a) H_2_SO_4_ (b) NaOH (c) NaNO_3_ (d) distilled water.

In this work, the adsorption/desorption experiments with different eluents (e.g., 0.1 M H_2_SO_4_, 0.1 M NaOH, 0.1 M NaNO_3_ and distilled water) were conducted to test the reusability of AC and FeAC for the removal of MB from aqueous systems (Fig 8). The results show that, FeAC has a strong potential to adsorb significant amount of MB molecules from the bulk as compared to its counterpart (AC) when applied in repeated cycles.

Fig 8 demonstrates the comparison of MB uptake by AC and FeAC up to 10 cycles using various eluents. The MB uptake by FeAC is significantly high (≥ 90%) and consistent in contrast to the spent AC. Among all of the desorbing agents used, the regeneration capacity of FeAC was more profound (90±5%) when H_2_SO_4_ was used as the desorbing agent/eluent due to the easy release of physisorbed dye molecules from the MB loaded adsorbent surface. Also, by using acidic desorbing agent, the surface of the adsorbent will receive more H^+^ ions from the solution and hence the cationic exchange of MB ions occur more rigorously. As discussed earlier in Section 3.2.1 (pH effect), at lower pH values the MB uptake is insignificant, hence the lower pH values corroborate the easy release of MB molecules from the FeAC surface under acidic conditions. Moreover, at lower pH values while treating with H_2_SO_4_ (as eluent), the surface of FeAC is allowed to have frequent interactions with H^+^ present in the bulk which triggers the surface protonation.

Comparatively higher MB desorption from the FeAC surface can be explained due to the protonation of FeAC surface which results in the electrostatic repulsion between MB molecules and the FeAC surface. In a similar study, Auta and Hameed [49] also reported a favorable desorption of MB from the MB loaded chitosan–clay composite material using an acidic desorbing agent (0.1 M HCl) and suggested a similar phenomenon.

**Fig 9:**
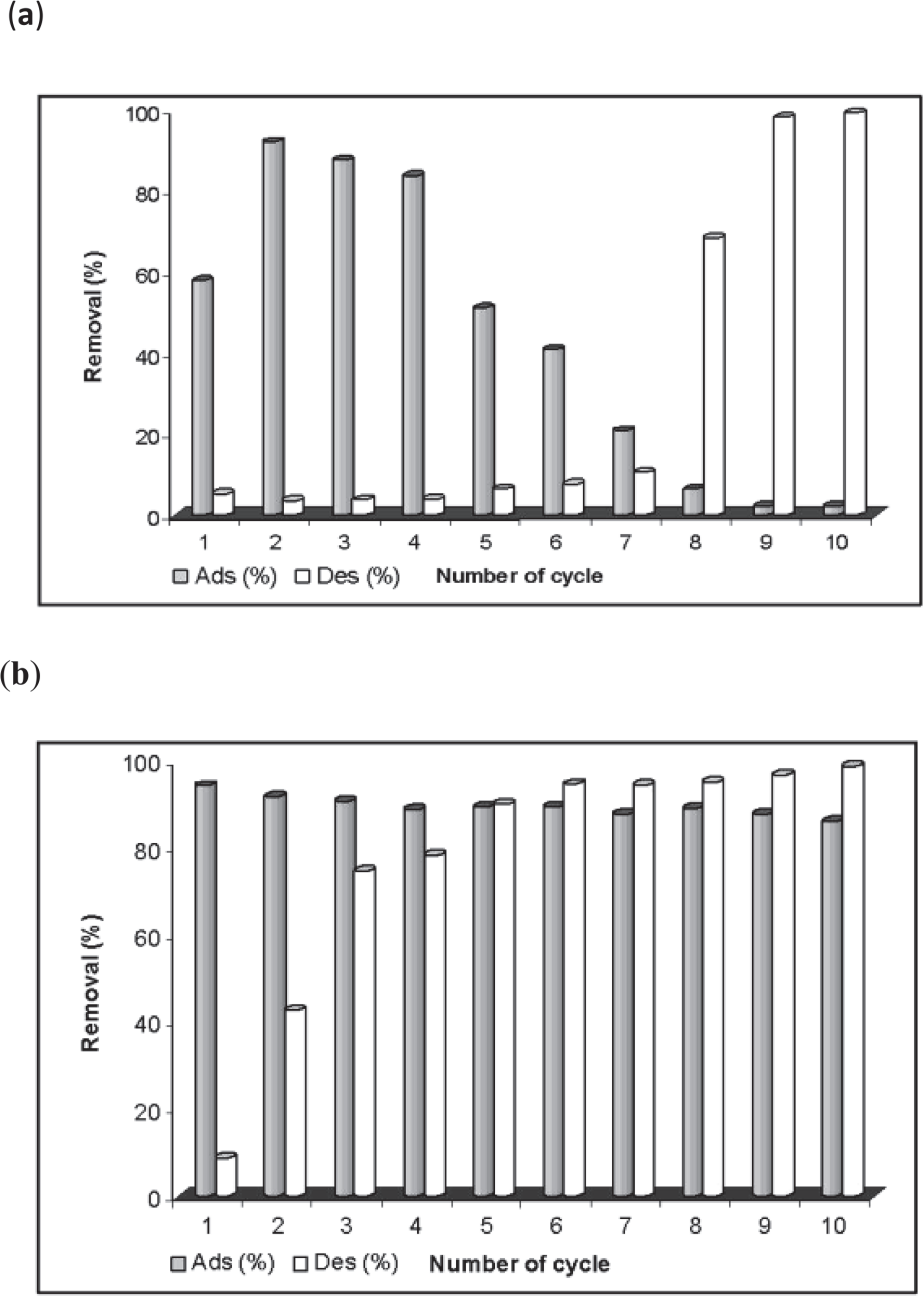
MB adsorption/desorption study on (a) AC and (b) FeAC by using H_2_SO_4_ as desorbing agent.

To regenerate the adsorbent after MB loading, desorption studies were conducted in detail. The results in Fig 8 show a relatively lower desorption of MB from the MB saturated AC surface during desorption process using an acidic desorbing agent, while the converse is true for FeAC. The desorption potential of AC was very low (below 10%) when 0.1 M H_2_SO_4_, 0.1 M NaOH or 0.1 M NaNO_3_ were used as eluents, which can be correlated with the poor reusability of AC. However, when distilled water was used as eluent, the MB desorption from AC was relatively high (up to 50%) in the first few cycles which then decreased to less than 10% after 10^th^ cycle. However, high desorption capacity in water by the MB loaded AC is non-beneficial for the practical usage in real dye polluted system. In contrast, the MB desorption from MB loaded FeAC surface was very little (< 10%) using distilled water. The MB molecules are adsorbed on the surface of AC from the bulk (MB solution) via a physisorption process (ΔG = -7.23 kJ/mol at 298 K) as reported in our earlier work [13]. Hence, it could be easily removed from the AC surface upon continuous shaking at 400 rpm for 3 h in distilled water. However, the interaction between FeAC with MB is slightly stronger than its counterpart (ΔG = -2.56 kJ/mol [13]), hence the MB desorption from FeAC in distilled water was minimized. Our findings are also in agreement with the reported literature where the desorption of dye from a dye loaded AC using distilled water as a desorbing agent refers to the weak interactions of the dye molecules with the surface of the adsorbent [50]. On the other side, a substantial percentage of MB was desorbed from the MB loaded FeAC surface (from 8 to 98% after 1^st^ to 10^th^ cycle, respectively) using 0.1 M H_2_SO_4_, as illustrated in Fig 9.

The negligibly small decrease, from 94.5 (cycle 1) to 86.3% (cycle 10), in the adsorption efficiency of FeAC during reproducibility studies, when 0.1 M H_2_SO_4_ was used as the desorbing agent can be correlated with the incomplete MB desorption from the surface of FeAC when repeatedly used. Another possible explanation could be the strong interactions of MB molecules with the active sites on FeAC due to some kind of chemical interactions at an inter molecular level hence limiting the desorption process. However, the overall regeneration ability of FeAC is relatively higher as compared to AC. It can be seen from Fig 9, that the adsorption–desorption potential of FeAC is more profound than its counterpart AC. The FeAC exhibits appreciably higher regeneration ability when used repeatedly in a batch system. The results also further demonstrate that FeAC has a strong adsorption potential and is more sustainable.

For AC, in the first 3 cycles of regeneration, the leaching of AC fine particles was observed as suspended solids in the analyte. Besides that, the AC surface was found to be nearly saturated after 5^th^ cycle and only a very small amount of MB was adsorbed in the next few cycles (see Fig 9). More specifically, from the 7^th^ to 10^th^ cycle the MB adsorption capacity of AC was reduced to less than 5% and this small amount could easily be removed from the surface, thus showing an abrupt increase in the desorption percentage in the last 3 cycles.

The regeneration capability of FeAC is of particular interest as it recovers the active sites for the dye molecules to be adsorbed and removed from the aqueous system upon reuse. These findings also show that the dye removal efficiency and the desorbability of FeAC is consistent (≥ 90±5%) when repeatedly used, but the case is less efficient for the AC. Previously, Wang et al. [29] used graphene–carbon nanotube composites for MB removal, but the removal efficiency of this material continuously decreased even in the first four cycles. However, in our case the recycle ability of our newly synthesized adsorbent is much higher and consistent for the removal of MB in the successive cycles. Thus, it can be proposed that FeAC is a potentially more stable and reusable adsorbent material and can be effectively used in a continuous flow system.

## Conclusions

The surface charge density (pH_*ZPC*_) of AC drastically decreased from 7.4 to 4.3, as a result of surface oxidation and iron impregnation, contributing to an increased acidic character of FeAC which is crucial for the significantly higher uptake of cationic dye (MB) from the aqueous system. The percentage of MB removal by FeAC was consistently high as compared to its counterpart (AC) in the entire range of pH (2–10), favorable at ambient temperature (298 K) and required a lower dosage (0.2 g showing up to 98% efficiency). Kinetic modeling indicates that pseudo–second order model explained the reaction mechanism well but the overall adsorption process was controlled by a number of processes such as film diffusion, pore diffusion and intraparticle diffusion. The feasibility and efficacy of the current work is correlated with the reusability studies of the spent adsorbents. Looking at the adsorption/desorption behavior of AC and FeAC, we can conclude that the FeAC is more suitable than the unmodified activated carbon to be used in a continuous flow system. The FeAC has shown a consistently high MB removal efficiency (up to 90%) when used repeatedly even after 10 cycles and this reflects on its high regeneration capability. The high adsorption/desorption efficiency of FeAC, highlight the practicability of the current findings and that FeAC is a promising adsorbent material for the removal of cationic dye pollutants from the wastewater.
